# Real-World Effectiveness, Safety, and Tolerability of Adjuvant CDK4/6 Inhibitor Therapy

**DOI:** 10.7759/cureus.117479

**Published:** 2026-10-05

**Authors:** Kajal Shah, Harsha P Panchal, Apurva Patel, Rajan Yadav

**Affiliations:** 1 Medical Oncology, The Gujarat Cancer &amp; Research Institute (Affiliated to B.J. Medical College), Ahmedabad, IND

**Keywords:** early breast cancer (ebc), endocrine therapy (et), hormone receptor-positive (hr+), human epidermal growth factor receptor 2-negative (her2-)-breast cancer, non-steroidal aromatase inhibitor (nsai)

## Abstract

Background: This study evaluates the real-world effectiveness, safety, and tolerability of adjuvant abemaciclib or ribociclib combined with a non-steroidal aromatase inhibitor (NSAI) in high-risk hormone receptor-positive (HR+), human epidermal growth factor receptor 2-negative (HER2-) early breast cancer in Western India.

Methods: We conducted a retrospective, single-institution study at The Gujarat Cancer and Research Institute (GCRI) in Ahmedabad, India, evaluating high-risk, stage II or III HR+/HER2- early breast cancer patients treated between November 2021 and May 2026. A total of 418 patients were evaluated across three groups: Group 1 received abemaciclib (150 mg twice daily for two years) plus NSAI (letrozole or anastrozole) (n = 138); Group 2 received ribociclib (400 mg daily, three weeks on, one week off for three years) plus NSAI (letrozole or anastrozole) (n = 138); and Group 3 received NSAI (letrozole or anastrozole) alone for ≥ 5 years (n = 142). The primary endpoint was invasive disease-free survival (iDFS), and secondary endpoints included overall survival (OS) and safety.

Results: Baseline characteristics were well-balanced across all three groups, with approximately three-quarters of the patients presenting with advanced stage III disease (77.8%, n=325/418). Adjuvant CDK4/6 inhibitor therapy significantly improved iDFS compared to NSAI alone. Abemaciclib plus NSAI (Group 1) was associated with an approximately 50% relative hazard reduction for an iDFS event compared to NSAI alone (HR 0.50; 95% CI, 0.27-0.91; p = 0.023). Ribociclib plus NSAI (Group 2) demonstrated a 48% relative hazard reduction compared to NSAI alone (HR 0.52; 95% CI, 0.29-0.93; p = 0.027). The estimated two-year iDFS rates were 93.5% (n = 129/138) in Group 1, 88.4% (n = 122/138) in Group 2, and 78.2% (n = 111/142) in Group 3. After applying a Bonferroni correction (α = 0.025), the iDFS benefit remained statistically significant for abemaciclib (p = 0.023), whereas ribociclib (p = 0.027) demonstrated a strong numerical trend that did not meet the adjusted threshold. Overall survival was not estimable due to insufficient events. Adverse events were consistent with established safety profiles: diarrhea was the most frequent adverse event in Group 1 (78.2%, n = 108/138), whereas neutropenia (63.0%, n = 87/138) and arthralgia (71.0%, n = 98/138) were prominent in Group 2. Permanent treatment discontinuation due to adverse events occurred in 13.8% (19/138) of patients in Group 1 and 13% (18/138) in Group 2.

Conclusion: In this retrospective non-randomized study, adding adjuvant abemaciclib or ribociclib to NSAI therapy was associated with two-year iDFS improvements in high-risk HR+/HER2- early breast cancer. However, after Bonferroni correction, the iDFS benefit remained statistically significant for abemaciclib and did not remain statistically significant for ribociclib. The therapies proved highly feasible and manageable in routine clinical practice.

## Introduction

Breast cancer is the most common cancer among Indian women, with hormone receptor-positive (HR+) (HR+) HER2-negative (HER2-) disease comprising approximately 70% to 75% of cases [1]. Standard management includes surgery, chemotherapy, and radiotherapy when indicated, followed by adjuvant endocrine therapy (ET) [2]. Although ET substantially reduces recurrence risk, clinically important late recurrences remain common, particularly in stage II/III disease [2, 3].

Adjuvant CDK4/6 inhibitors have, therefore, become an important strategy for selected high-risk patients. In monarchE, abemaciclib 150 mg twice daily for two years plus ET improved five-year iDFS compared with ET alone (83.6% vs 76.0%; HR 0.68) [4, 5]. NATALEE demonstrated a similar invasive disease-free survival (iDFS) benefit with ribociclib 400 mg daily on a three-weeks-on, one-week-off schedule for three years plus ET, with a three-year iDFS of 90.4% versus 87.1% [6]. In contrast, PALLAS and PENELOPE-B did not demonstrate significant iDFS improvement with adjuvant palbociclib, supporting a regimen-specific rather than uniform class effect [7, 8].

Randomized trials may differ from routine practice in patient selection, monitoring, and treatment delivery. We therefore conducted a retrospective comparative study at Gujarat Cancer Research Institute (GCRI), Ahmedabad, India, evaluating adjuvant abemaciclib or ribociclib with non-steroidal aromatase inhibitor (NSAI) versus NSAI-based ET alone in high-risk stage II/III HR+ human epidermal growth factor receptor 2-negative (HER2-) early breast cancer. The study aims to evaluate the real-world effectiveness, safety, and tolerability of adjuvant abemaciclib or ribociclib with NSAI therapy, with iDFS as the primary endpoint and overall survival (OS)/safety as secondary endpoints. 

## Materials and methods

Study design and patient population

This retrospective single-institution study was conducted at the GCRI in Ahmedabad, Western India. We evaluated medical records of patients diagnosed with stage II or III, histologically confirmed HR+/HER2- early breast cancer who were treated in routine clinical practice between November 2021 and May 2026.

Eligible patients met the following inclusion criteria: age ≥ 18 years, female or male sex, confirmed estrogen receptor (ER) and/or progesterone receptor (PR) positive and HER2- disease, and classified as having high-risk disease. High-risk disease was defined using strict clinical and pathological parameters matching standard guidelines: either ≥ 4 positive pathologic axillary lymph nodes or 1 to 3 positive axillary lymph nodes combined with at least one additional high-risk factor (tumor size ≥ 5 cm, or histologic grade 3 disease). All included patients had undergone surgical treatment (modified radical mastectomy or breast-conserving surgery) and had received neoadjuvant or adjuvant chemotherapy, as well as adjuvant postoperative radiotherapy if clinically indicated.

Patients were excluded from the analysis if they presented with metastatic disease (stage IV), occult breast cancer, inflammatory breast cancer, or if they had incomplete treatment records or follow-up data.

Treatment groups and dosing guidelines

Eligible patients were categorized into three treatment groups based on the adjuvant therapeutic regimen they received: Group 1, abemaciclib 150 mg twice daily for two years plus NSAI letrozole 2.5 mg daily or anastrozole 1 mg daily (n=138); Group 2, ribociclib 400 mg daily for three weeks followed by one week off for three years plus NSAI letrozole 2.5 mg daily or anastrozole 1 mg daily (n=138); and Group 3, letrozole 2.5 mg or anastrozole 1 mg daily for ≥5 years (n=142). Premenopausal women and men received ovarian function suppression with leuprolide 3.75 mg every 28 days.

Statistical analysis

Time-to-event outcomes (iDFS and OS) were estimated using the Kaplan-Meier method, and survival curves were compared using the log-rank test. Cox proportional hazards regression models were constructed to calculate HRs and their corresponding 95% CI for comparative treatment effects, adjusting for key baseline characteristics. All statistical tests were two-sided, with statistical significance defined as p < 0.05. Statistical analyses were performed utilizing standard software, IBM SPSS Statistics version 32 (IBM Corporation, Armonk, NY, USA).

## Results

Baseline demographic and clinical characteristics

A total of 418 patients meeting the high-risk eligibility criteria were included and evaluated in this retrospective study: 138 received abemaciclib plus standard ET NSAI (Group 1), 138 received ribociclib plus standard ET NSAI (Group 2), and 142 received ET NSAI alone (Group 3).

The baseline clinical and pathological characteristics of the study groups were well-balanced across the three treatment groups (Table 1), with no statistically or clinically meaningful imbalances observed. Median age was 48.0 years (range, 28-77 years) in Group 1, 48.0 years (range, 28-77 years) in Group 2, and 46.0 years (range, 34-67 years) in Group 3. Most patients were under 65 years of age, with 81.2% (n = 112/138) in Group 1, 87.7% (n = 121/138) in Group 2, and 81.7% (n = 116/142) in Group 3 being younger than 65 years. The vast majority of patients were female (99.3%, 100%, and 98.6% across Groups 1, 2, and 3, respectively). Furthermore, over 90% of patients across all groups had Eastern Cooperative Oncology Group (ECOG) performance scores of 0 or 1, and ER-positive status was confirmed in over 92% of the cases.

**Table 1:** Baseline demographic and clinicopathological characteristics of the study groups (N = 418) Abbreviations: ECOG, Eastern Cooperative Oncology Group; ER, estrogen receptor; ET, endocrine therapy; NOS, not otherwise specified; PR, progesterone receptor; MRM, modified radical mastectomy; BCS, breast conservative surgery; Pos, positive; Neg, negative. Values are presented as numbers and percentages.

Category/Parameter	Abemaciclib + ET (Group 1) (N = 138)	Ribociclib + ET (Group 2) (N = 138)	ET Alone (Group 3) (N = 142)
Age, n (%)			
<65 years	112 (81.2%)	121 (87.7%)	116 (81.7%)
>65 years	26 (18.8%)	17 (12.3%)	26 (18.3%)
Sex, n (%)			
Male	1 (0.7%)	0 (0.0%)	2 (1.4%)
Female	137 (99.3%)	138 (100.0%)	140 (98.6%)
ECOG Status, n (%)			
0	78 (56.5%)	82 (59.4%)	86 (60.5%)
1	58 (42.0%)	52 (37.7%)	54 (38.0%)
2	2 (1.5%)	4 (2.9%)	2 (1.5%)
Hormonal Status, n (%)			
Estrogen Receptor Pos	134 (97.1%)	128 (92.8%)	133 (93.7%)
Estrogen Receptor Neg	4 (2.9%)	10 (7.2%)	9 (6.3%)
Progesterone Receptor Pos	78 (56.5%)	68 (49.3%)	84 (59.2%)
Progesterone Receptor Neg	60 (43.5%)	70 (50.7%)	58 (40.8%)
Pathological Stage, n (%)			
Stage I	1 (0.7%)	2 (1.5%)	2 (1.4%)
Stage IIA	17 (12.3%)	14 (10.1%)	15 (10.6%)
Stage IIB	12 (8.7%)	14 (10.1%)	16 (11.2%)
Stage IIIA	45 (32.6%)	43 (31.2%)	42 (29.6%)
Stage IIIB	15 (10.9%)	19 (13.8%)	21 (14.8%)
Stage IIIC	48 (34.8%)	46 (33.3%)	46 (32.4%)
Histopathology, n (%)			
Invasive Ductal (NOS)	129 (93.5%)	127 (92.0%)	132 (92.9%)
Invasive Lobular	3 (2.2%)	4 (2.9%)	3 (2.1%)
Medullary	2 (1.4%)	3 (2.1%)	4 (2.8%)
Papillary	1 (0.7%)	2 (1.5%)	1 (0.7%)
Mucinous	3 (2.2%)	2 (1.5%)	2 (1.4%)
Postoperative Radiotherapy	118 (85.5%)	121 (87.7%)	129 (90.6%)
Prior Chemotherapy, n (%)			
Neoadjuvant Chemotherapy	68 (49.3%)	72 (52.2%)	81 (57.0%)
Anthracyclines	66 (47.8%)	68 (49.3%)	79 (55.6%)
Taxanes	64 (46.4%)	64 (46.4%)	77 (54.2%)
Anthracyclin and Taxanes	64 (46.4%)	64 (46.4%)	76 (53.5%)
Adjuvant Chemotherapy	70 (50.7%)	66 (47.8%)	61 (42.9%)
Anthracyclines	68 (49.3%)	64 (46.4%)	59 (41.5%)
Taxanes	66 (47.8%)	62 (44.9%)	58 (40.8%)
Anthracyclin and Taxanes	64 (46.3%)	60 (43.5%)	57 (40.1%)
Surgical Resection, n (%)			
BCS	66 (47.8%)	64 (46.4%)	69 (48.6%)
MRM	72 (52.2%)	74 (53.6%)	73 (51.4%)
Endocrine Agent, n (%)			
Letrozole	136 (98.5%)	135 (97.8%)	137 (96.5%)
Anastrozole	2 (1.5%)	3 (2.2%)	5 (3.5%)
Ovarian Suppression, n (%)	34 (24.6%)	38 (27.5%)	42 (29.6%)

Crucially, the group represents an exceptionally high-risk clinical population. Approximately three-quarters of the patients presented with advanced Stage III disease (78.3% n = 108/138 in Group 1, 78.3% n = 108/138 in Group 2, and 76.8% n = 109/142 in Group 3). Specifically, the sub-stages IIIA, IIIB, and IIIC were distributed as follows: Group 1 (32.6% IIIA, 10.9% IIIB, 34.8% IIIC); Group 2 (31.2% IIIA, 13.8% IIIB, 33.3% IIIC); and Group 3 (29.6% IIIA, 14.8% IIIB, 32.4% IIIC). The majority of patients had invasive ductal carcinoma (not otherwise specified) and had received prior postoperative radiotherapy: 85.5% (n = 118/138) in Group 1, 87.7% (n = 121/138) in Group 2, and 90.6% (n = 129/142) in Group 3. Standard chemotherapy exposure was high, with approximately half of the patients receiving neoadjuvant chemotherapy (Group 1: 49.3%, Group 2: 52.2%, Group 3: 57.0%) and the remainder receiving adjuvant chemotherapy. Letrozole was the main NSAI utilized across all three groups. Because approximately 50% of the study population underwent neoadjuvant chemotherapy prior to surgical resection, these Stage I pathologic findings (ypStage I) represent tumors that underwent pathological downstaging from their initial baseline Stage II or III clinical presentation.

Safety and tolerability profiles

Adjuvant treatment with CDK4/6 inhibitors was associated with distinct, manageable toxicity profiles that were highly consistent with known safety data. In Group 1 (abemaciclib + NSAI), the primary non-hematological toxicity was diarrhea, which occurred in 78.2% (n=108/138) of patients (any grade; 4.3% Grade 3, 1.4% Grade 4). Other common toxicities included thrombocytopenia (38.4%), neutropenia (34.0%), anemia (18.1%), alopecia (20.2%), vomiting (17.3%), and urinary tract infections (18.1%). Grade 3/4 thrombocytopenia and neutropenia were reported in 14.4% and 17.3% of patients, respectively.

In Group 2 (ribociclib + NSAI), the toxicity profile was characterized by hematological and constitutional toxicities, as well as joint symptoms. Neutropenia was highly prominent, occurring in 63.0% (n=87/138) of patients (any grade; 3.6% Grade 3, 1.4% Grade 4). Arthralgia was exceptionally frequent in Group 2, occurring in 71.0% (n=98/138) of patients (27.5% Grade 3, 3.6% Grade 4). Fatigue was also highly prevalent, affecting 49.2% (n=68/138) of patients (16.6% Grade 3, 3.6% Grade 4). Additionally, transaminase elevations were more prominent in the ribociclib cohort, with alanine aminotransferase (ALT) increased in 19.5% of patients (2.8% Grade 3, 4.3% Grade 4) and aspartate aminotransferase (AST) increased in 31.8% of patients (1.4% Grade 3, 2.2% Grade 4) (Table 2).

**Table 2:** Documented adverse events stratified by treatment group and CTCAE severity (N = 418) Abbreviations: ALT, alanine aminotransferase; AST, aspartate aminotransferase; CTCAE, Common Terminology Criteria for Adverse Events; ET, endocrine therapy; G3, Grade 3; G4, Grade 4. Values are presented as numbers and percentages.

Adverse Event Category	Abemaciclib + ET (G1) (N=138)			Ribociclib + ET (G2) (N=138)			ET Alone (G3) (N=142)		
	Any grade	G3	G4	Any grade	G3	G4	Any grade	G3	G4
Diarrhea	108 (78.2%)	6 (4.3%)	2 (1.4%)	7 (5.0%)	0 (0%)	0 (0%)	11 (7.7%)	1 (0.7%)	0 (0%)
Thrombocytopenia	53 (38.4%)	15 (10.8%)	5 (3.6%)	69 (50.0%)	2 (1.4%)	0 (0%)	6 (4.2%)	2 (1.4%)	1 (0.7%)
Neutropenia	47 (34.0%)	18 (13.0%)	6 (4.3%)	87 (63.0%)	5 (3.6%)	2 (1.4%)	4 (2.8%)	1 (0.7%)	1 (0.7%)
Anemia	25 (18.1%)	4 (2.8%)	1 (0.7%)	37 (26.8%)	4 (2.8%)	1 (0.7%)	6 (4.3%)	2 (1.4%)	1 (0.7%)
Arthralgia	22 (15.9%)	6 (4.3%)	4 (2.8%)	98 (71.0%)	38 (27.5%)	5 (3.6%)	77(54.2%)	43(30.2%)	21(14.7%)
Nausea	11 (7.9%)	3 (2.1%)	0 (0%)	15 (10.8%)	4 (2.8%)	2(1.4%)	8 (5.6%)	2 (1.4%)	0 (0%)
Vomiting	24 (17.3%)	6 (4.3%)	0 (0%)	12 (8.6%)	5 (3.6%)	1 (0.7%)	6 (4.2%)	1 (0.7%)	1 (0.7%)
Abdominal pain	11 (7.9%)	8 (5.7%)	1 (0.72%)	7 (5.0%)	3 (2.1%)	0 (0%)	5 (3.5%)	0 (0%)	1 (0.7%)
Alopecia	28 (20.2%)	7 (5.0%)	4 (2.8%)	9 (6.5%)	4 (2.8%)	2 (1.4%)	14 (9.8%)	2 (1.4%)	1 (0.7%)
Headache	14 (10.1%)	4 (2.8%)	0 (0%)	10 (7.2%)	2 (1.4%)	0 (0%)	8 (5.6%)	0 (0%)	0 (0%)
Fatigue	24 (17.3%)	5 (3.6%)	1 (0.72%)	68 (49.2%)	23 (16.6%)	5 (3.6%)	12 (8.4%)	2 (1.4%)	1 (0.7%)
Constipation	11 (7.9%)	3 (2.1%)	0 (0%)	5 (3.6%)	1 (0.72%)	0 (0%)	6 (4.2%)	1 (0.7%)	0 (0%)
Hot flushes	14 (10.1%)	4 (2.8%)	1 (0.72%)	27 (19.5%)	6 (4.3%)	3 (2.2%)	12 (8.4%)	1 (0.7%)	1 (0.7%)
Asthenia	13 (9.4%)	3 (2.12%)	0 (0%)	45 (32.6%)	12 (8.6%)	5 (3.6%)	65 (45.7%)	8 (5.6%)	6 (4.2%)
ALT increased	14 (10.1%)	7 (5.0%)	2 (1.4%)	27 (19.5%)	4 (2.8%)	6 (4.3%)	13 (9.1%)	6 (4.2%)	1 (0.7%)
AST increased	16 (11.5%)	9 (6.5%)	2 (1.4%)	44 (31.8%)	2 (1.4%)	3 (2.2%)	23 (16.1%)	8 (5.6%)	2 (1.4%)
Upper Resp. Inf.	24 (17.3%)	4 (2.8%)	1 (0.72%)	23 (16.6%)	4 (2.8%)	1 (0.7%)	12 (8.4%)	2 (1.4%)	1 (0.7%)
Urinary Tract Inf.	25 (18.1%)	6 (4.3%)	1 (0.72%)	13 (9.4%)	3 (2.2%)	2 (1.4%)	5 (3.5%)	1 (0.7%)	0 (0%)

Importantly, standard NSAI alone (Group 3) was primarily associated with typical endocrine class effects: arthralgia was recorded in 54.2% (n=77/142) of patients (any grade; 30.2% Grade 3, 14.7% Grade 4) and asthenia in 45.7% (any grade; 5.6% Grade 3, 4.2% Grade 4). Age-related degenerative joint disease also contributed to reported arthralgia.

Treatment compliance and feasibility were remarkably high in these real-world groups. Permanent treatment discontinuation of the CDK4/6 inhibitor due to unacceptable toxicities or adverse events was low, occurring in 13.8% (n = 19/138 ) of patients receiving abemaciclib (Group 1) and 13% (n = 18/138) of patients receiving ribociclib (Group 2). The remaining patients successfully managed toxicities via standard supportive medications, clinical monitoring, and temporary dose interruptions or modifications. The detailed comparative toxicity spectrum is tabulated in Table 2, with key hallmark toxicities demonstrated in Figure 1.

**Figure 1:**
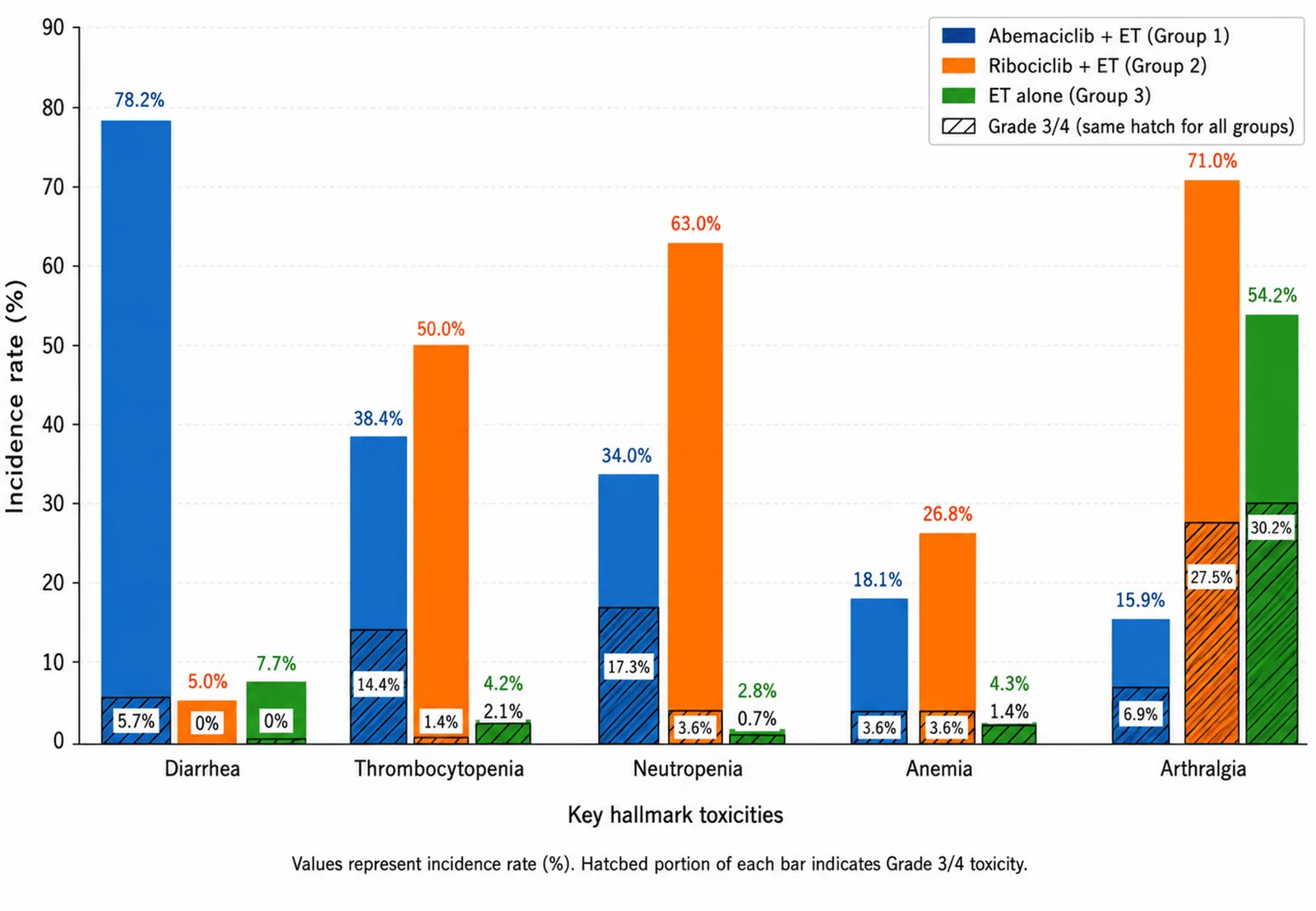
Selected adverse event rates (any grade versus Grade 3/4) highlighting distinct safety profiles of the treatment cohorts. The same hatch pattern (//) denotes Grade 3/4 toxicity across all treatment groups. ET: endocrine therapy

iDFS outcomes

Adjuvant therapy combining a CDK4/6 inhibitor with standard NSAI therapy was associated with a statistically and clinically significant survival advantage. At two years, estimated iDFS rates reached 93.5% in Group 1 (abemaciclib + NSAI) and 88.4% in Group 2 (ribociclib + NSAI), compared with 78.2% in Group 3 (NSAI alone). 

Cox proportional hazards regression models revealed highly substantial relative reductions in the hazard of an iDFS recurrence event for patients receiving combination CDK4/6 inhibitors: Group 1 (abemaciclib + NSAI) exhibited an approximately 50% relative reduction in the hazard of an iDFS event compared with Group 3 (ET alone) (HR, 0.50; 95% CI, 0.27-0.91; p = 0.023). Group 2 (ribociclib + NSAI) exhibited an approximately 48% relative reduction in the hazard of an iDFS event compared with Group 3 (ET alone) (HR, 0.52; 95% CI, 0.29-0.93; p = 0.027).

When applying a conservative Bonferroni correction (adjusted significance threshold alpha = 0.025), the comparison for abemaciclib remains statistically significant (p = 0.023), whereas the comparison for ribociclib (p = 0.027) falls marginally above the adjusted threshold.

The overall Kaplan-Meier comparison across all three treatment curves was highly statistically significant (log-rank p < 0.05), indicating consistent and robust survival improvements from the addition of either CDK4/6 inhibitor. These primary efficacy findings are graphically summarized in Figure 2.

**Figure 2:**
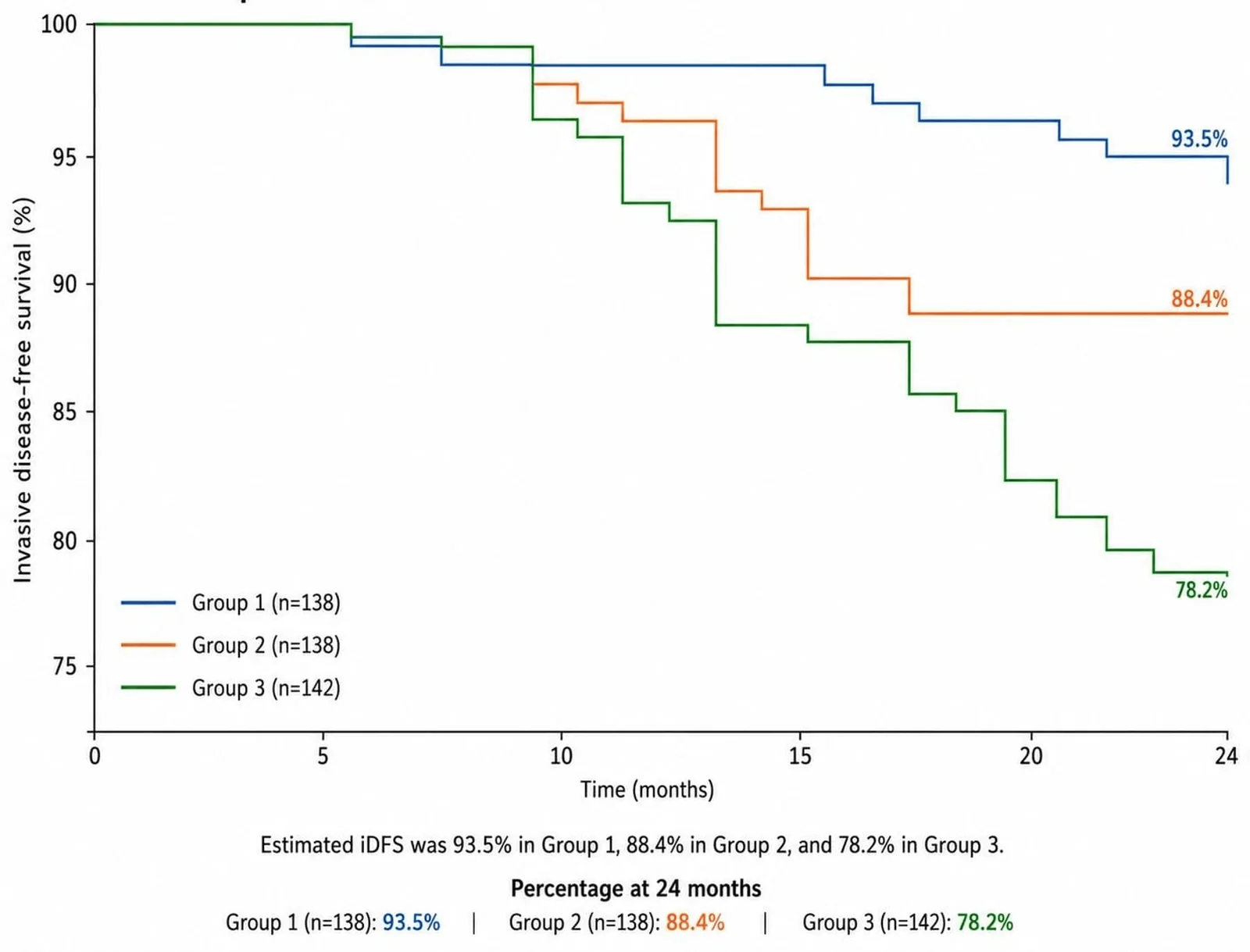
Kaplan-Meier curves for invasive disease-free survival (iDFS) of Group 1, abemaciclib plus endocrine therapy (N = 138); Group 2, ribociclib plus endocrine therapy (N = 138); Group 3, endocrine therapy alone (N = 142).

OS data were not estimable due to short median follow-up. Long-term follow-up is ongoing to establish whether these early iDFS improvements will translate into a significant overall survival benefit.

iDFS events occurred in 56 of 418 patients (13.4%) across the study cohort. By treatment arm, iDFS events were reported in 9 of 138 patients (6.5%) in Group 1 (abemaciclib + NSAI), 16 of 138 patients (11.6%) in Group 2 (ribociclib + NSAI), and 31 of 142 patients (21.8%) in Group 3 (NSAI alone).

## Discussion

The present retrospective real-world study evaluated the effectiveness and tolerability of adjuvant CDK4/6 inhibitor therapy in patients with high-risk HR+/HER2- early breast cancer treated in routine clinical practice in Western India. The addition of either abemaciclib or ribociclib to ET NSAI was associated with improved iDFS compared with ET NSAI alone. At two years, the estimated iDFS rates were 93.5% with abemaciclib plus NSAI, 88.4% with ribociclib plus NSAI, and 78.2% with ET NSAI alone. In adjusted analyses, the hazard of an iDFS event was approximately half that observed with ET NSAI alone, with adjusted HRs of 0.50 for abemaciclib and 0.52 for ribociclib. These findings provide supportive real-world evidence that the clinical benefit observed with adjuvant CDK4/6 inhibitors in randomized trials may also be observed in routine oncology practice in an Indian population.

The findings are consistent with the results of the monarchE trial. In the updated analysis of monarchE, adjuvant abemaciclib combined with ET produced a sustained improvement in iDFS compared with ET alone, with a five-year iDFS rate of 83.6% versus 76.0% and a HR of 0.68 [4].

The results also complement evidence from the NATALEE trial evaluating ribociclib in combination with ET. The initial analysis demonstrated a significant improvement in iDFS with ribociclib plus ET compared with ET alone, with three-year iDFS rates of 90.4% and 87.1%, respectively [6]. More recently, the four-year analysis confirmed the persistence of this benefit, with iDFS rates of approximately 88.5% with ribociclib plus ET compared with 83.6% with ET alone [9].

An important feature of the present study was the substantial burden of advanced-stage disease. Approximately three-quarters of patients had stage III disease, including patients with stage IIIA, IIIB, and IIIC tumors. This represents a clinically high-risk population and differs from the broader spectrum of patients enrolled in NATALEE, which included selected patients with node-negative stage II disease [6,9]. The findings of the present study support the feasibility of implementing these therapies in appropriately selected high-risk patients in routine Indian practice. Real-world factor include advanced disease burden at presentation, may contribute to lower baseline survival compared with clinical trial populations.

The safety findings were broadly consistent with the known toxicity profiles of the two agents. Diarrhea was the most frequently observed adverse event with abemaciclib, whereas neutropenia and other hematologic toxicities were more prominent with ribociclib. Arthralgia and fatigue were also commonly observed, while elevations in alanine aminotransferase and aspartate aminotransferase were more frequent with ribociclib [6]. These findings are consistent with the safety profiles reported in the randomized trials and reinforce the importance of regular hematologic and biochemical monitoring during treatment.

The relatively low rates of permanent treatment discontinuation observed in this study suggest that both treatment strategies were feasible in routine clinical practice when accompanied by appropriate toxicity surveillance and dose modification. Real-world studies of adjuvant abemaciclib have similarly emphasized the importance of early identification and management of adverse events, treatment interruption when necessary, and dose modification to maintain treatment persistence [10,11].

The study provides comparative real-world outcome data for both currently used adjuvant CDK4/6 inhibitors alongside an ET NSAI-only group in western India.

Limitations

This study has limitations. First, its retrospective study with non-randomized treatment allocation, missing Ki-67 data, and a relatively small sample size. Second, the follow-up period was relatively short, and OS data were immature at the time of analysis. Third, the retrospective, non-randomized design introduces inherent susceptibility to selection bias and residual confounding.

## Conclusions

This retrospective non-randomized real-world study demonstrated that the addition of adjuvant abemaciclib or ribociclib to NSAI significantly improved two-year iDFS among patients with high-risk HR+/HER2- stage II or III early breast cancer in Western India. Both adjuvant CDK4/6 inhibitor therapies, when combined with NSAI therapy, were associated with improvements in iDFS compared with NSAI alone in high-risk HR+/HER2- early breast cancer. After applying Bonferroni correction, the iDFS improvement remained statistically significant for abemaciclib, whereas ribociclib did not remain statistically significant. The toxicities observed were consistent with known clinical profiles and proved manageable, with low discontinuation rates reflecting excellent clinical compliance. These findings provide vital real-world validation supporting the routine clinical use of adjuvant CDK4/6 inhibitors to prevent recurrence in this high-risk population.
